# Highly Pathogenic Avian Influenza Virus (H5N1) Infection in Red Foxes Fed Infected Bird Carcasses

**DOI:** 10.3201/eid1412.080470

**Published:** 2008-12

**Authors:** Leslie A. Reperant, Geert van Amerongen, Marco W.G. van de Bildt, Guus F. Rimmelzwaan, Andrew P. Dobson, Albert D.M.E. Osterhaus, Thijs Kuiken

**Affiliations:** Princeton University, Princeton, New Jersey, USA (L.A. Reperant, A.P. Dobson); Erasmus Medical Centre, Rotterdam, the Netherlands (G. van Amerongen, M.W.G. van de Bildt, G.F. Rimmelzwaan, A.D.M.E. Osterhaus, T. Kuiken)

**Keywords:** Highly pathogenic avian influenza virus, H5N1, red fox, vulpes, carnivore, encephalitis, crossing of the species barrier, research

## Abstract

Eating infected wild birds may put wild carnivores at high risk for infection with highly pathogenic avian influenza (HPAI) virus (H5N1). To determine whether red foxes (*Vulpes vulpes*) are susceptible to infection with HPAI virus (H5N1), we infected 3 foxes intratracheally. They excreted virus pharyngeally for 3–7 days at peak titers of 10^3.5^–10^5.2^ median tissue culture infective dose (TCID_50_) per mL and had severe pneumonia, myocarditis, and encephalitis. To determine whether foxes can become infected by the presumed natural route, we fed infected bird carcasses to 3 other red foxes. These foxes excreted virus pharyngeally for 3–5 days at peak titers of 10^4.2^–10^4.5^ TCID_50_/mL, but only mild or no pneumonia developed. This study demonstrates that red foxes fed bird carcasses infected with HPAI virus (H5N1) can excrete virus while remaining free of severe disease, thereby potentially playing a role in virus dispersal.

## Abstract 2

Foxes experimentally fed infected bird carcasses excrete virus for >5 days without exhibiting severe disease and may thus disperse the virus.

Influenza A viruses rarely infect species of the order Carnivora. However, since 2003, highly pathogenic avian influenza (HPAI) viruses of subtype H5N1 have infected a wide range of carnivore species. Within the past 30 years, and before the emergence of HPAI viruses (H5N1), 5 documented outbreaks of influenza virus infections occurred in 2 carnivore species—the harbor seal (*Phoca vitulina*) (*1*–*4*), and the American mink (*Mustela vison*) (*5*). In both species, the infection resulted in respiratory disease. In addition, influenza virus infection has been detected by virus culture or serologic examination in other carnivores, namely, domestic dogs (*Canis lupus familiaris*) (*6*,*7*), domestic cats (*Felis catus*) (*8*,*9*), and bears kept in captivity (species not stated) (*9*); however, these animals did not show clinical signs of disease. Also, recently, outbreaks of equine influenza virus (H3N8) infections resulted in respiratory disease in domestic dogs (*10*,*11*). In contrast, within the past 5 years, HPAI viruses (H5N1) have infected and killed carnivores belonging to 7 species: captive tigers (*Panthera tigris*) and leopards (*P. pardus*) (*12*,*13*); domestic cats (*14*–*17*); captive Owston’s palm civets (*Chrotogale owstoni*) (*18*); a domestic dog (*19*); a free-living stone marten (*Martes foina*) (*20*); and a free-living American mink (*21*). In these species, the infection resulted in both respiratory and extrarespiratory lesions, demonstrating systemic infection beyond the respiratory system. The most frequently reported clinical signs for all species were respiratory distress, neurologic signs, or both.

The sources of most HPAI virus (H5N1) infections in carnivores were traced to infected birds eaten by the animals (*12*–*15*,*19*). Until 2005, carnivores infected with HPAI virus (H5N1) were either wild carnivores kept in captivity or domestic carnivores that ate infected domestic or peridomestic birds (*12*–*14*,*19*). Since 2005, and after the spread of HPAI virus (H5N1) of the Qinghai sublineage (clade 2.2) outside Southeast Asia in poultry and wild bird populations, carnivores infected with HPAI virus (H5N1) included for the first time free-living wild carnivores, which presumably ate infected wild birds (*20*,*21*).

The occurrence of HPAI viruses (H5N1) in wild bird populations is likely to result in the exposure and infection of free-living wild carnivore species. In particular, abundant and widespread species of wild carnivores that have opportunistic feeding habits and that feed on wild birds may be at high risk for exposure. The red fox (*Vulpes vulpes*) is one of the most abundant and widespread species of wild carnivores in Eurasia. Partly because of rabies eradication (*22*,*23*), fox populations in western Europe have increased drastically since the mid-1980s (e.g., >140% in Germany; *24*). The red fox is also an opportunistic carnivore species and has a diverse diet, which includes small mammals and birds (*24*,*25*). Therefore, it may likely hunt or scavenge wild birds infected with HPAI viruses (H5N1). However, the susceptibility of this species to infection with influenza viruses is unknown.

In this study, we asked 2 questions: 1) Are red foxes susceptible to infection with a wild bird isolate of HPAI virus (H5N1) from clade 2.2? and 2) Can red foxes become infected by the presumed natural route of infection, i.e., after feeding on infected bird carcasses? To answer these questions, we experimentally assessed the excretion pattern (based on route, duration, and concentration of virus excretion) and pathogenicity (based on clinical signs, death rates, and distribution of lesions and virus) of a wild bird isolate of clade 2.2 HPAI virus (H5N1) in red foxes infected intratracheally and in red foxes fed infected bird carcasses.

## Materials and Methods

### Virus Preparation

A virus stock was prepared of influenza virus A/whooper swan/Germany/R65-1/2006 (H5N1), which was isolated from a wild whooper swan (*Cygnus cygnus*) found dead on Rügen Island, Germany, in February 2006. (The isolate was kindly provided by Dr Martin Beer, Friedrich-Loeffler-Institute, Greifswald–Insel Riems, Germany.) It was propagated twice in MDCK cells and titrated according to standard methods (*26*). The stock reached an infectious virus titer of 10^6.9^ median tissue culture infectious dose (TCID_50_) per mL. It was then diluted in phosphate-buffered saline (PBS) to obtain a concentration of 10^4^ TCID_50_/mL.

### Experimental Design

Eight juvenile (6–10 months of age) red foxes were obtained from a control program involving the fox population in the Netherlands. All were negative for antibodies against influenza viruses according to a commercially available nucleoprotein-based ELISA test (European Veterinary Laboratory, Woerden, the Netherlands) and for antibodies against canine distemper virus according to a virus neutralization assay. The foxes had been treated against helminthic infections with 50 mg of fenbendazole when they were 2.5 months old and with 22.7 mg of praziquantel, 22.7 mg of pyrantel base as pyrantel pamoate, and 113.4 mg of febantel 2 months later. One month before the start of the experiment, transponders (Star-Oddi, Reykjavik, Iceland) that record body temperature every 15 minutes were placed in the peritoneal cavity of each fox, after the animal had been anesthetized with intramuscular injections of ketamine (5 mg/kg) and medetomidine (0.05 mg/kg). During the experiment, 6 foxes (nos. 1–3 and 5–7) were singly housed in negatively pressurized isolation units. Two negative-control foxes (nos. 4 and 8) were housed separately in an indoor enclosure.

To determine susceptibility to infection, we infected 3 foxes (nos. 1–3) intratracheally with a clade 2.2 HPAI virus (H5N1). Each anesthetized fox received 2.5 × 10^4^ TCID_50_ of virus in a volume of 2.5 mL through a catheter. One anesthetized fox (no. 4) was sham-infected intratracheally with 2.5 mL of PBS and served as a negative control. To determine whether red foxes can become infected by the presumed natural route of infection, we fed infected birds to 3 foxes (nos. 5–7). The infected birds were 1-week-old chicks that had been infected intratracheally with 2.5 × 10^4^ TCID_50_ of the HPAI virus (H5N1) in a volume of 0.5 mL. At 24 hours postinoculation, the chicks were euthanized by cervical dislocation and fed to foxes nos. 5–7 (2 whole chicks/fox). Homogenates of liver, lung, kidney, and brain from infected chicks contained 10^6.3^ to >10^9.3^ TCID_50_/g tissue; pharyngeal and cloacal swabs reached titers of 10^4.5^ to 10^7.2^ TCID_50_/mL. On the basis of the relative weight of the lungs, liver, kidneys, and brain of 1-week-old chicks weighing 50 to 55 g (*27*,*28*), foxes fed 2 chick carcasses received a minimal titer of 10^10^ TCID_50_. Virus titers in internal organs of dead wild or domestic birds naturally infected with HPAI virus (H5N1) have been reported sparingly; however, an article from Japan reported high virus titers, e.g., as high as 10^7.5^ TCID_50_/mL, in the lung of a naturally infected large-billed crow (*Corvus macrorhynchos*) (*29*). High titers were also detected in internal organs of 10-week-old chickens and in highly susceptible species of wild swans, geese, and ducks that were experimentally infected with a clade 2.2 HPAI virus (H5N1), e.g., whooper swans, mute swans (*Cygnus olor*), bar-headed geese (*Anser indicus*), common pochards (*Aythya ferina*), and tufted ducks (*Aythya fuligula*) (*30*–*32*). For example, virus titers in internal organs of common pochards and tufted ducks infected with a low dose of clade 2.2 HPAI virus (H5N1) reached >10^6^ TCID_50_/mL (*32*). Our experimental design thus likely reproduces natural exposure after ingestion of dead or moribund birds infected with the virus. Our negative control was 1 fox (no. 8) that was fed 2 whole chicks that had been sham infected with PBS.

Before inoculation and at 1, 2, 3, 5, and 7 days postinoculation (dpi), all foxes were anesthetized with ketamine-medetomidine, after which they were weighed, and nasal, pharyngeal, and rectal swabs were collected and placed in 3 mL of virus transport medium (Hank’s balanced salt solution containing 10% glycerol, 200 U/mL penicillin, 200 μg/mL streptomycin, 100 U/mL polymyxin B sulfate, and 250 μg/mL gentamicin). Each day, foxes were observed for clinical signs; observers were ≈2 m from the isolation units. At 7 dpi, all foxes were anesthetized with ketamine-medetomidine and euthanized by exsanguination. Experimental procedures were approved by an independent animal care and use committee.

### Postmortem and Immunohistochemical Examinations

Necropsy examinations and tissue sample collection were performed according to a standard protocol. After fixation in 10% neutral-buffered formalin and embedding in paraffin, tissue sections were stained with hematoxylin and eosin for histologic evaluation, or they were processed according to an immunohistologic method that used a monoclonal antibody against the nucleoprotein of influenza A virus as a primary antibody for detection of influenza viral antigen (*33*). Lung tissue of an experimentally infected cynomolgus macaque (*Macaca fascicularis*) experimentally infected with influenza virus A/Hong Kong/156/97 (H5N1) served as a positive control. Negative controls were created by omitting the primary antibody or replacing the primary antibody with an irrelevant antibody, immunoglobulin G2 (clone 20102; R&D, Abingdon, UK). The following tissues were examined by these 2 methods: conjunctiva, nasal concha, nasal septum, trachea, lung (6 specimens/fox), tongue, esophagus, stomach, duodenum, jejunum, ileum, cecum, colon, tonsil, tracheobronchial lymph node, mesenteric lymph node, spleen, thymus, heart, liver, pancreas, kidney, adrenal gland, urinary bladder, olfactory bulb, cerebrum (at level of hippocampus), cerebellum, and brain stem.

### Virus Titrations

The same tissues examined for histopathologic changes were also sampled for viral titration. Tissue samples were weighed and homogenized in 3 mL of transport medium with a homogenizer (Kinematica Polytron, Lucerne, Switzerland). Serial dilutions (10-fold) of the tissue homogenates and swabs were inoculated into MDCK cells in triplicate as described previously (*26*). The minimal detectable titer was 10^0.8^ TCID_50_/mL. All experiments were performed under BioSafety Level 3 conditions.

## Results

### Clinical Signs

Clinical signs were not observed in foxes infected intratracheally or in foxes fed infected bird carcasses. However, body temperature of 2 of the 3 foxes infected intratracheally (nos. 1 and 2) and of 1 of the 3 foxes fed infected bird carcasses (no. 5) rose from 38.5°C–39°C (reference range) to 40°C–40.5°C at 2 to 4 dpi. No clinical signs and no rise in body temperature were observed for the negative-control foxes (nos. 4 and 8).

### Virology

**Figure 1:**
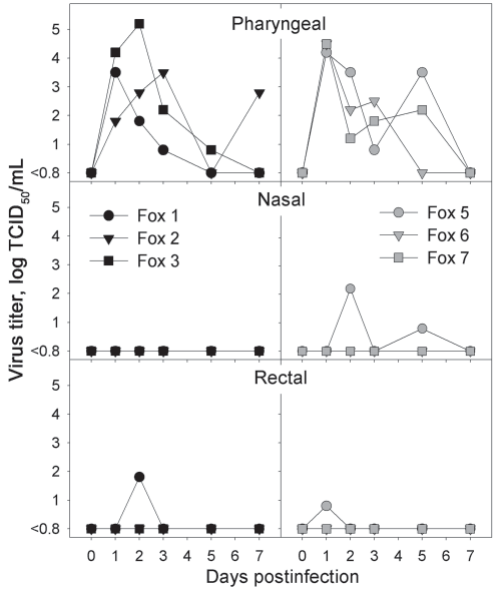
Infectious virus titers obtained from pharyngeal, nasal, and rectal swabs of foxes infected intratracheally with highly pathogenic avian influenza (HPAI) virus (H5N1) (left, black symbols) or fed chicks infected with HPAI virus (H5N1) (right, gray symbols) at various time points after infection. No virus was isolated from any swabs of the negative-control foxes. TCID_50_, median tissue culture infectious dose.

The virus was isolated from pharyngeal swabs from all infected foxes and from nasal and rectal swabs from 1 fox and rectal swabs from another (Figure 1). Foxes infected intratracheally excreted the virus through the pharynx from 1 dpi on, up to 3–7 dpi; peak titers of pharyngeal excretion were 10^3.5–^10^5.2^ TCID_50_/mL at 1–3 dpi. Foxes that had been fed infected bird carcasses excreted the virus through the pharynx from 1 dpi on, up to 3–5 dpi; peak titers of pharyngeal excretion were 10^4.2^–10^4.5^ TCID_50_/mL at 1 dpi. Student *t* test showed no significant difference in the patterns of pharyngeal excretion between the 2 groups of foxes according to areas under the curve (*t* = –0.667, df = 4, p = 0.54). No virus was isolated from any swabs from any negative-control foxes.

The virus was isolated from the trachea (10^2.6^ TCID_50_/g tissue) and lung (10^3.3^ TCID_50_/g) of 1 of 3 foxes infected intratracheally (no. 2), and from the tonsil (10^2^ TCID_50_/g) of another fox infected intratracheally (no. 3). No virus was isolated from any of the organs of the foxes fed infected bird carcasses or of the negative-control foxes.

### Gross Examination

**Figure 2:**
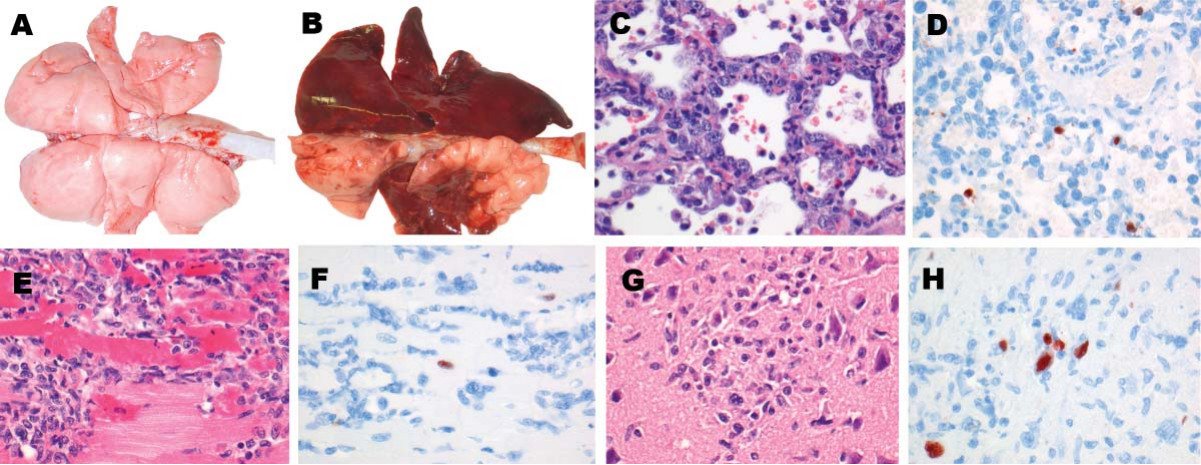
Lesions and associated expression of influenza virus antigen in respiratory and extrarespiratory organs of foxes infected intratracheally with HPAI virus (H5N1), at 7 days postinoculation. A) Lungs of control fox sham-inoculated with phosphate-buffered saline. B) Lungs of intratracheally inoculated fox presenting extensive consolidated lesions (darkened areas), characterized by C) diffuse alveolar damage and regeneration (type II pneumocyte hyperplasia) and D) expression of influenza virus antigen in the nucleus and, to a lesser extent, cytoplasm of mononuclear and epithelial cells. E) Focus of inflammation and cardiomyocytic necrosis in the heart, associated with F) expression of influenza virus antigen in the nucleus of cardiomyocytes. G) Focus of gliosis and neuronal necrosis in the cerebrum, associated with H) expression of influenza virus antigen in the nucleus and, to a lesser extent, cytoplasm of glial cells and neurons. Panels C–H, original magnification ×40.

Of the 3 foxes infected intratracheally, 2 (nos. 1 and 2) had extensive multifocal or coalescing pulmonary lesions, which were dark purple and slightly firm (Figure 2). The estimated percentage of affected lung tissue was 20% (no. 1) and 80% (no. 2). In contrast, 1 of the 3 foxes infected intratracheally (no. 3) and all foxes fed infected bird carcasses had >2 small multifocal lesions (1–5 mm), which affected <5% of the lungs. In addition, 2 of the 3 foxes fed infected bird carcasses (nos. 5 and 6) had randomly distributed petechial hemorrhages throughout the lungs. Moderate enlargement of the spleen, tonsils, and/or tracheobronchial lymph nodes was observed in all foxes, whether infected intratracheally or fed infected bird carcasses. Negative-control foxes had no respiratory or extrarespiratory lesions.

### Histopathologic Findings

Histologic lesions were found in foxes infected intratracheally and in foxes fed infected bird carcasses. However, the lesions were more severe in foxes infected intratracheally (Table). The 2 most severely affected foxes (nos. 1 and 2, infected intratracheally) had severe hemorrhagic bronchointerstitial pneumonia with extensive coalescing lesions of inflammation and necrosis, characterized by macrophage and neutrophil infiltration of the alveolar walls and loss of histologic architecture. The alveolar and bronchiolar lumina were filled with alveolar macrophages, neutrophils, and erythrocytes, mixed with fibrin and cellular debris. In both foxes, sloughing of the alveolar and bronchiolar epithelia indicated necrosis, and type II pneumocyte and bronchiolar epithelial hyperplasia indicated regeneration (Figure 2). The other foxes (no. 3, infected intratracheally, and all foxes fed infected bird carcasses) had minimal to mild bronchointerstitial or interstitial pneumonia. They had small- to medium-sized foci of inflammation in the lungs, located mostly around the bronchioles and characterized by thickened alveolar walls that were infiltrated with macrophages and neutrophils. Type II pneumocyte and bronchiolar epithelial hyperplasia was observed in the lungs of fox no. 7. Respiratory organs of negative-control foxes had no lesions.

**Table:** Distribution of lesions and influenza virus antigen expression in experimentally infected red foxes*

Route of inoculation, fox no.	Lesions				Influenza virus antigen		
	Lungs	Heart	Brain		Lungs	Heart	Brain
Intratracheal inoculation							
1	+++	–	+		–	–	–
2	+++	+	++		++	+	+++
3	++	–	–		–	–	–
Fed infected bird carcasses							
5	+	–	–		–	–	–
6	–	–	–		–	–	–
7	+	–	–		–	–	–

*Foxes were infected either intratracheally with highly pathogenic avian influenza (HPAI) virus (H5N1) or by being fed chicks infected with HPAI virus (H5N1); they were examined at 7 days postinoculation. Respiratory lesions, extrarespiratory lesions, or influenza virus antigen expression were not observed in negative-control foxes. –, absence of lesions (no cells expressing the influenza virus antigen); +, mild and focal or multifocal lesions (few cells expressing the influenza virus antigen); ++, moderate and multifocal lesions (moderate number of cells expressing the influenza virus antigen); +++, severe and extensive lesions (numerous cells expressing the influenza virus antigen).

Extrarespiratory histologic lesions were seen only in foxes infected intratracheally, namely, in the heart of fox no. 2 and in the cerebrum of foxes nos. 1 and 2. Fox no. 2 had multiple inflammatory and necrotic lesions in the myocardium, characterized by infiltration of macrophages and neutrophils and necrotic cardiomyocytes (Figure 2). The cerebrum of foxes nos. 1 and 2, infected intratracheally, had multiple lesions of acute to subacute encephalitis, from mild to severe, characterized by perivascular cuffing, foci of gliosis or neuronal necrosis, or a combination of these lesions; their cerebellum and brain stem did not show any lesions (Figure 2). No relevant lesions were seen in other organs, including organs of the digestive tract, of any other foxes.

### Immunohistochemical Findings

Cells expressing the influenza virus antigen were present in the lungs, heart, and brain of 1 of 3 foxes infected intratracheally (no. 2) but in none of the foxes fed infected bird carcasses (Table). Mononuclear cells and alveolar epithelial cells in damaged parts of the lungs expressed the influenza virus antigen as diffuse red staining in their nucleus and, to a lesser extent, in their cytoplasm. Occasional cardiomyocytes in the periphery of a lesion in the heart expressed the influenza virus antigen as granular red staining in their nucleus. Lastly, neuronal and glial cells in the periphery of lesions in the cerebrum expressed the influenza virus antigen as granular to diffuse red staining in their nucleus and, to a lesser extent, their cytoplasm (Figure 2). No influenza virus antigen was detected in any cells of other organs, including the intestinal tract, of any other foxes.

## Discussion

This study demonstrates that red foxes are susceptible to infection with a wild bird isolate of HPAI virus (H5N1) from clade 2.2. Red foxes can become infected after eating infected bird carcasses, and they can excrete the virus for as many as 5 days in the absence of severe disease. Therefore, naturally infected red foxes may potentially survive infection in the wild and excrete and disperse HPAI viruses (H5N1) within their home ranges. The size of foxes’ home ranges depends on the environmental conditions and availability of food resources, but typically it varies between 1 km² and 10 km² (*34*). Red foxes are highly mobile and may travel 5–20 km within their home range during a night (*35*). A juvenile fox traveled 90 km in 1 direction within 5 days during fall dispersal from its place of birth (*35*). Furthermore, red foxes have colonized most urbanized areas in Europe, resulting in increased contact with domestic and peridomestic animals (*23*). They may transmit the virus to domestic species, such as poultry. Therefore, we propose that this abundant and widespread carnivorous species be surveyed for exposure to or infection with HPAI viruses (H5N1) in influenza-endemic areas or in areas experiencing outbreaks of HPAI virus (H5N1) infections in wild bird populations. Where foxes are hunted, carcasses may be routinely sampled and tested. Where foxes are protected or not hunted, live trapping, bleeding, and pharyngeal swabbing of anesthetized foxes may be implemented.

Although red foxes fed infected bird carcasses may survive infection, severe pneumonia, myocarditis, and encephalitis may develop in those inoculated intratracheally. Frequent findings of HPAI virus (H5N1) infections in naturally infected carnivores were pneumonia associated with respiratory distress and encephalitis (in some cases associated with neurologic signs) (*12*–*14*,*16*,*18*–*21*). In most instances, the animals were either euthanized because of the severity of the disease or were found dead. Surprisingly, foxes with severe respiratory and cerebral lesions did not show any visible clinical signs. Foxes, being wild animals, were wary in the presence of humans and changed their behavior even when observed from a distance. This behavior may have prevented us from observing subtle clinical signs, notably abnormal breathing. A cat that died of HPAI virus (H5N1) infection in Germany did not show visible clinical signs 24 hours before death, despite marked respiratory lesions (*16*), which suggests that even severe respiratory lesions may not be noticed clinically. Clinical manifestations of neurologic lesions in infected foxes may have gone unnoticed because the lesions were in the cerebrum rather than in the cerebellum. Although cerebellar lesions may cause conspicuous neurologic signs, e.g., ataxia and loss of balance, cerebral lesions may cause more subtle clinical signs, e.g., altered mental attitude, which were not noticed under these experimental conditions (*36*).

Foxes may exhibit more severe disease after eating infected birds under natural conditions than under the controlled conditions of our feeding experiments, because of poorer health, possible co-infections, and poorer nutritional status of wild animals. For instance, the cats that died of HPAI virus (H5N1) infection in Germany were all infected with *Aelurostrongylus* spp., and pulmonary aelurostrongylosis was considered to have contributed to the severity of the disease in these animals (*16*). Fatal cases of HPAI virus (H5N1) infection in red foxes may have been missed after the spread of HPAI viruses (H5N1) in poultry and wild bird populations outside Asia because fox carcasses are difficult to locate and because those found may likely be routinely tested for rabies and canine distemper rather than for influenza virus infection. Therefore, we suggest that red foxes with neurologic signs or red foxes found moribund or dead in disease-endemic areas or in areas experiencing outbreaks of HPAI virus (H5N1) infections in wild bird populations be tested for HPAI virus (H5N1) infection.

Foxes infected intratracheally and those fed infected bird carcasses exhibited similar virus-shedding patterns despite the different routes of exposure and despite marked differences in the severity and extent of associated lesions. Foxes fed infected bird carcasses likely inhaled virus particles during mastication. The differences in the severity and extent of associated lesions may have thus resulted from a difference in the respiratory inoculum received by foxes infected intratracheally and those fed infected carcasses. We did not observe influenza antigen–positive neuronal cells in the submucosal or myenteric plexi of the small intestine of foxes fed infected bird carcasses. This contrasts with findings in cats infected in the same way; in the cats, these cells were positive, which suggests the intestine as a route of virus entry (*37*). The fact that the viral shedding patterns were similar despite the marked differences in severity and extent of respiratory lesions is surprising and difficult to explain. On the basis of the absence of influenza antigen–positive cells in the respiratory tract, all but 1 fox appeared to have cleared the virus from this site by 7 dpi. Signs of regeneration in bronchiolar and alveolar epithelia were observed both in foxes infected intratracheally and in those fed infected bird carcasses.

In summary, we have shown that red foxes are susceptible to infection with a wild bird isolate of HPAI virus (H5N1) from clade 2.2, can become infected after feeding on infected bird carcasses, and can excrete the virus for as many as 5 days without severe disease developing. Surveillance and monitoring of HPAI virus (H5N1) infections may therefore be beneficially expanded to red foxes, and potentially to other free-living wild carnivores, in influenza-endemic areas and in areas experiencing outbreaks of HPAI virus (H5N1) infections in wild bird populations.
